# A Development Architecture for Serious Games Using BCI (Brain Computer Interface) Sensors

**DOI:** 10.3390/s121115671

**Published:** 2012-11-12

**Authors:** Yunsick Sung, Kyungeun Cho, Kyhyun Um

**Affiliations:** 1 The Department of Computer & Information Science & Engineering, University of Florida, Gainesville, FL 32601, USA; E-Mail: sung@ufl.edu; 2 Department of Multimedia Engineering, Dongguk University-Seoul, 26 Pildong, 3 Ga, Jung-gu, Seoul 100-715, Korea; E-Mail: khum@dongguk.edu

**Keywords:** BCI (brain–computer interface) sensors, BCI serious games, BCI framework, brainwaves

## Abstract

Games that use brainwaves via brain–computer interface (BCI) devices, to improve brain functions are known as BCI serious games. Due to the difficulty of developing BCI serious games, various BCI engines and authoring tools are required, and these reduce the development time and cost. However, it is desirable to reduce the amount of technical knowledge of brain functions and BCI devices needed by game developers. Moreover, a systematic BCI serious game development process is required. In this paper, we present a methodology for the development of BCI serious games. We describe an architecture, authoring tools, and development process of the proposed methodology, and apply it to a game development approach for patients with mild cognitive impairment as an example. This application demonstrates that BCI serious games can be developed on the basis of expert-verified theories.

## 1. Introduction

In general, serious games differ from purely entertainment games in that they attempt to achieve specific learning goals. Serious games that use brain–computer interface (BCI) devices are known as BCI serious games [1]. The development of BCI serious games is more difficult than that of other entertainment games [1], and is also different from that of other BCI applications [2]. For instance, BCI serious game development requires expert knowledge of brainwave features and brain functions, which entertainment game developers do not usually have. This makes it difficult to develop BCI serious games. In comparison to BCI serious games, BCI applications have, in most cases, been developed for specific users. For example, the application may control a wheelchair [2] or measure the progress of dementia in a patient [3,4]. However, BCI serious games are targeted at a range of individuals who have access to BCI devices. Thus, they must support a variety of BCI devices and should not depend on a specific platform. Furthermore, BCI applications detect user's brain status by identifying a certain pattern in the measured brainwaves. The identified patterns are then applied to the BCI application. However, BCI serious games use brainwaves as a game controller. For example, the direction in which a game character moves is determined based on the measured brainwaves. In this case, a process is required for controlling the game in a fair way using diverse brainwaves from each user.

To consider the specialty of BCI serious games, the following three problems should be considered: first, the necessary understanding of brainwave features and brain functions should be minimized, as this can take considerable burdens to develop. It is difficult for entertainment game developers to effectively develop BCI serious games for their users. Thus, a number of projects cannot be started because of a lack of qualified experts or due to cost problems. To minimize the knowledge required by the game developers, experts' knowledge should be described in such a way that entertainment game developers can easily understand and apply it to BCI serious games. Second, BCI serious games measure brainwaves using BCI functions provided by a diverse range of third parties, and game developers may spend a lot of time supporting diverse BCI devices. Thus, various devices should be controlled by a unified interface to reduce development time. Third, it is very difficult to use brainwaves to control BCI serious games, because each user will have a different distribution of brainwaves. For example, some users' brainwaves will be measured to have bigger amplitude than others. Therefore, we require a process that normalizes individual characteristics, and then transfers the brainwaves to the signal for the BCI serious game. Fourth, a systematic BCI serious game development process is needed for experts and game developers dealing with brain functions and brainwaves. This will allow diverse developers to quickly and easily produce games together.

The existing research on BCI application development has generally dealt with approaches to extract features from brainwaves, rather than the functions required for game development. For example, BCI++, BCI2000, and Open-ViBE provide a variety of functions to process brainwaves, such as their measurement and the identification of different features [5]. Some researchers have integrated 3D engines to develop 3D games. However, these studies have not provided solutions to the following diverse requirements for BCI serious game development. First, it is difficult for entertainment game developers to apply brainwaves to games because they usually have an insufficient understanding of brainwave features and brain functions. Consequently, the BCI-related development process should be separated from the BCI serious game development and handled by experts in the field of brainwaves. Second, the above-mentioned frameworks (with the exception of Open-ViBE) do not usually support cheap BCI devices suitable for BCI serious games. Generally, the BCI devices used for BCI applications are expensive multi-channel equipment to accurately analyze the brainwaves of a user. However, BCI serious games may use low price BCI devices to enable more users to access them. Such devices include Emotiv EPOC and NeuroSky Mindset, although their accuracy is relatively low compared with more expensive devices. In some cases, a number of functions cannot be used because the BCI devices used in BCI serious games are different from those used in BCI applications. Third, the above researches have not provided a transform function to allow the measured brainwaves to be used as the game control signals.

Some researches into the use of the measured brainwaves in BCI serious games have transformed the brainwaves, thus generating signals with a uniform distribution for each user [6,7]. Other researches have controlled diverse BCI devices using a unified interface, which transforms the brainwaves into signals suitable for BCI serious games [8]. However, in order to intuitively develop BCI serious games whilst reducing the time and cost required, we need a method that handles expert knowledge, diverse authoring tools for repetitive processes, and a BCI serious game development methodology.

BCI serious games can be more accessible and interesting than other therapies or rehabilitation exercises. They can provide effective therapies or preventive measures for patients who find it difficult to concentrate, or children who do not wish to undertake therapy. Moreover, BCI serious games can also include functions to prevent dementia or activate the brain for general users. Easier BCI serious game development will enable more games to be developed.

In this paper, we propose a methodology for BCI serious game development. Firstly, we describe the roles involved in producing a BCI serious game. Next, to simplify the development process, we define templates by separating those parts requiring expert input from the overall development process, and use a diverse range of authoring tools to develop BCI serious games based on these templates.

This paper is organized as follows: Section 2 describes the BCI engines and various BCI toolkits. Section 3 introduces our approach for BCI serious game development, and Section 4 gives a detailed description of the proposed approach. In Section 5, we discuss the results of our BCI serious game development methodology, and we finally, draw our conclusions in Section 6.

## 2. Related Work

Research into BCI engines and toolkits for BCI serious games is still in its infancy. In this section, we review some research that can be applied to BCI serious game development. Most BCI application methods have been developed for research purposes. For example, Open-ViBE provides the functionality for processing measured brainwaves, and measures electroencephalography (EEG) and magnetoencephalography (MEG) [9]. Open-ViBE removes noise and intensifies signals during the preprocessing step. This involves extracting and classifying feature vectors, and translating them into commands. BCI2000 consists of a Source Module, Signal Processing Module, User Application Module, and Operator Module [10]. The Source Module provides data measurement and storage functions, and the Signal Processing Module extracts and translates features. The User Application Module is developed by the user. The Operator Module provides reference system parameters for each module. Unlike other frameworks, BCI2000 does not have a preprocessing step. BioSig is a software library for biomedical signal-processing tools [11]. It provides diverse functions for handling brainwaves based on various platforms. In particular, it provides functions for extracting and classifying features from brainwaves. Moreover, BioSig also incorporates visualization functions. The BCI++ framework has been proposed for laboratory implementation and daily life applications [12]. It is composed of a Hardware Interface Module (HIM) and AEnima. HIM includes functions for collecting and recording measured brainwaves, and AEnima provides the user interface, including graphics and audio engines. AEnima is installed on the subject's PC and HIM on the operator's PC. The operator analyzes changes in brainwaves corresponding to the subject's AEnima activity. BCI++ is open source (based on C++). BCI++ contains two modules, each of which provides an independent interface. Thus, it is not suitable for games that are implemented as one independent application.

Some researchers have proposed approaches for processing brainwaves in order to develop BCI serious games. For example, there have been researches into the use of measured brainwaves as the control signal [6,7]. One divides the measured brainwaves into multiple sections, makes same distribution per section to remove user-dependent features, and uses them as the game signals [6]. Another approach transforms the measured brainwaves into signals with a normalized distribution [7]. These studies provide the functions to remove the features of a user from the measured brainwaves, and control the BCI serious game in a fair way per user. The interface research enables BCI devices developed by diverse third parties to be controlled by a unified interface [8]. This enables BCI serious game developers to reduce the time needed to deal with BCI devices by unifying the functions that measure brainwaves, and provides functions for converting brainwaves and using them as game control signals, and allows for the creation of new signals by mixing brainwaves. Moreover, the framework provides an approach for simultaneously applying both functions to a BCI serious game.

Various BCI frameworks and functions to handle brainwaves have been proposed. However, to develop BCI serious games, they require functions that consider their unique characteristics. In this paper, we propose a methodology that utilizes frameworks and authoring tools to reduce the burden of development. This methodology provides BCI serious game functions and simplifies the development process.

## 3. BCI Serious Game Development Approach

BCI serious games are developed on the basis of a variety of research results, including those related to brainwaves, neurofeedback, cognition training, and brain functions. In this section, we concisely explain the concepts behind a variety of approaches to BCI serious game development.

### 3.1. Hybrid Neurofeedback Model

**Figure 1.**
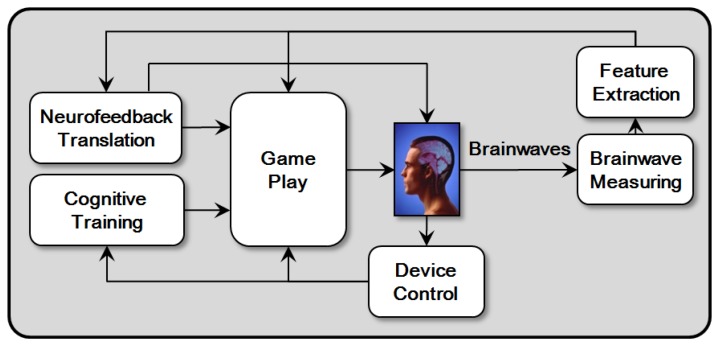
Hybrid Neurofeedback Model. This model contains neurofeedback and cognitive training.

Research into neurofeedback has already been utilized in various fields, where its effects have been verified. Our goal is to enhance the traditional neurofeedback effects. Therefore, we propose a model that also applies cognitive training [13,14] to BCI serious games with neurofeedback [15,16] to improve the effects of brain functions, as shown in Figure 1. When users play a BCI serious game, they can improve their brains by not only utilizing neurofeedback but also applying cognitive training simultaneously. We call this model Hybrid Neurofeedback Model (HNM).

We apply this model to the development of BCI serious games. Users control BCI serious games directly through extracted features or input devices, and utilize the output of neurofeedback translation by a BCI device directly for the next measurement. In other words, Users play a BCI serious game with cognitive training by touching game items on touchscreen or manipulating them with a mouse or keyboard through Device Control module. User's activities produce the results of cognitive functions represented by the game. Cognitive Training module reflects them on the game. Moreover, Brainwave Measuring module measures the brainwaves of users while they play the game, and Feature Extraction module identifies certain features. Each feature is transferred to Neurofeedback Translation module, which is also reflected on the game. User sees status changes of the game through Game Play module. As a result, the processes and results of neurofeedback and cognitive training can be confirmed through BCI serious games.

### 3.2. Classification of Roles and Developers

**Figure 2.**
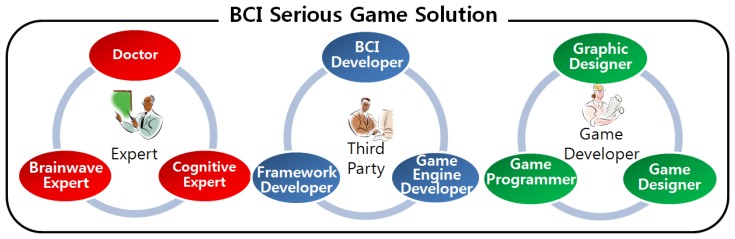
Developers related to BCI Serious Game Development. There are experts, game developers, and third parties.

BCI serious games are produced by various developers. When BCI serious games are first developed, there are many difficulties with the roles of developers and processes of BCI serious game development. So, methodical development processes are required. For these, the type of developers should be defined, and their roles should be assigned. Figure 2 shows various developers and the relationship among them.

In our approach, we define developers with knowledge of brainwave, brain functions, and cognitive training as experts. Each expert is either a doctor, or brainwave expert, or cognitive expert. For example, doctors may have technical knowledge of brain functions, brainwave experts may be able to analyze the relationship between technical knowledge and brainwaves, and cognitive experts may focus on the design and configuration of cognitive training in order to combine the optimum elements depending on the user's condition. BCI serious game development requires diverse experts to cooperate in the design of brainwave types, processes, and processing methods to improve brain function. By separating the development processes of experts from the other development processes, the amount of expert knowledge required by the other developers could be minimized. Table 1 describes the role of the experts in developing a BCI serious game. We have integrated the roles of all experts to define one BCI serious game development guide, because brain functions, brainwaves, and cognitive training are closely related. These experts define the types of brainwaves, frequency of measurement, and processing order required for BCI serious game development. Experts usually describe an early version of templates which entertainment game developers easily understand. The composition and process of these templates will be described below.

Game development of both entertainment games and BCI serious games requires a variety of engines and toolkits, such as networks and 3D engines. As the game developers cannot code all of the engines and toolkits, they use many existing utilities. Unlike entertainment games, BCI serious games require toolkits to process brainwaves, BCI devices and BCI drivers to measure brainwaves, as well as engines. For example, BCI framework developers provide the middleware to apply the brainwaves to a game using other BCI devices and drivers. Table 2 shows the output from third parties. We exclude such works as listed in Table 2, from our BCI-related development process.

Game developers from the entertainment field, do not usually have any knowledge about BCI serious games. To reduce the development overhead of BCI serious game, we had better ask game developers to get additional knowledge for BCI serious game development. Game developers include graphic designers, game designers, and game programmers. Game designers configure games based on advice from experts, and game programmers code the games in accordance with the designs prepared by the game designers. Graphic designers draw images and build 3D models through general graphics tools. These works will be integrated to BCI serious games. Because their drawing process is the same as for entertainment games, we omit the explanation on the graphic design process. Table 3 shows the output from the game developers. The significant difference compared to entertainment game development is that BCI serious games are designed according to the expert-defined templates.

### 3.3. Templates

Templates are defined with order-based symbols. Each symbol is connected to other symbols according to the time at which they are invoked. The symbols are divided into BCI symbols and flow symbols. BCI symbols contain data that are needed to control brainwaves, such as the type of brainwaves used for a BCI serious game. BCI symbols are only defined by experts as shown in Table 4. There are two BCI symbols: one for initializing BCI and the other for measuring brainwaves.

Flow symbols are utilized to describe the game process, which is defined by experts and game designers. The different flow symbols are defined in Table 5. All templates are designed by connecting symbols sequentially. Templates are divided into two types: an expert template and a game design template. Expert templates and game design templates include BCI symbols and flow symbols. Therefore, the processes that require expert knowledge are separated. In addition, only expert templates set and change the parameters of BCI symbols. The flow charts described by the experts and the game designers are integrated, rather than existing independently. Firstly, the experts create and define templates before the game designers modify the defined templates.

Experts define expert templates through a series of meetings. The expert templates describe the development approach in a way that can be easily understood without assuming that game developers have technical knowledge. All experts participate in defining this template, and determine when a BCI device should be initialized, utilized, and terminated. The types of brainwaves, frequency, and process order required for playing a game are defined by a symbol-based flow chart. The game design template defines how to play a game using measured brainwaves. The game designers define the game play, using flow charts to describe the game's processes.

### 3.4. BCI Serious Game Development Process

BCI serious games are developed based on templates. We propose diverse authoring tools for supporting the definition and utilization of templates. First, an expert authoring tool is provided to enable experts to describe the templates, which ensures the experts know what needs to be defined for serious game development. Only the expert authoring tool has the ability to create new templates. This prevents the development of BCI serious games that are not verified by experts.

Second, a game design authoring tool is provided to enable game designers to design the game based on expert templates. Accordingly, game designers concentrate solely on game design. This tool provides the functions for describing the flow of playing the game. Game designers can select a template depending on the game they are developing, and design the game.

Third, a programmer authoring tool is provided to load the game design template and generate game codes and modules based on the game design template. Thus, programmers can only develop in accordance with the given designs. The generated game code and modules are inserted into the program source code developed by the game programmers. The programmers complete the game using the generated game codes and game modules, as well as BCI devices, BCI drivers, a BCI framework, a network engine, and a 3D engine.

**Figure 3.**
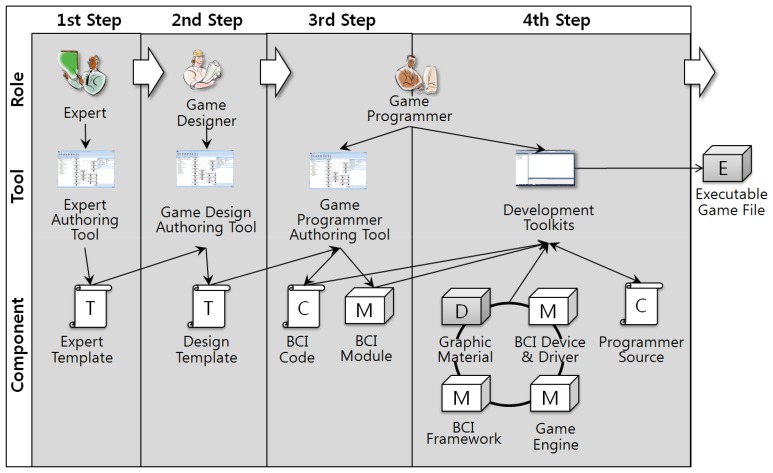
Relationships between Developers, Authoring Tools, and Outputs. There are three kinds of authoring tool that generate diverse outputs.

Figure 3 illustrates the development processes when using authoring tools. First, experts define their templates using the expert authoring tool. The game designers load the defined templates, design the game by modifying the loaded templates, and then store the designed templates as game design templates. After storing the templates, game programmers load the templates and generate BCI codes and BCI modules using the game programmer authoring tool. Finally, game programmers develop the BCI serious game using the generated BCI codes and BCI modules. The BCI game runs with BCI devices, BCI drivers, a BCI framework, game engines and graphical materials.

## 4. BCI Solution Development

By integrating various strands of the game development, we complete a BCI serious game. This section describes the development of a BCI solution using BCI authoring tools, which are based on HNM.

### 4.1. BCI Solution Architecture

**Figure 4.**
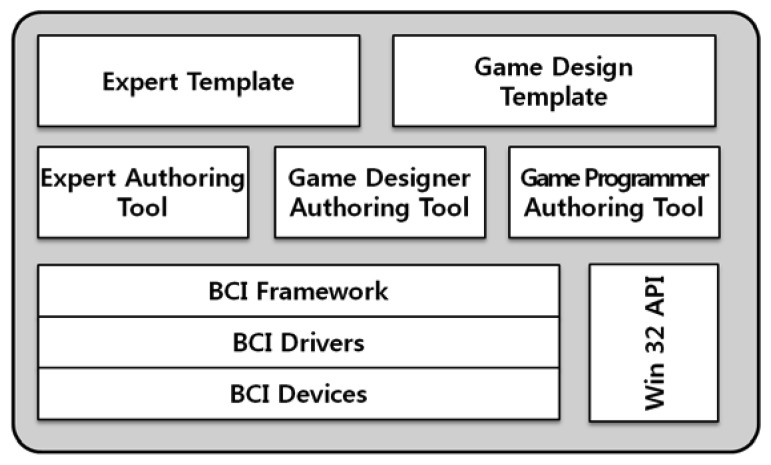
BCI solution architecture of hybrid neurofeedback model. The BCI solution provides the authoring tools for a BCI serious game, which game programmers develop by using the outputs of authoring tools.

The BCI solution is an environment for developing a BCI serious game. This solution includes BCI devices, BCI drivers, the BCI framework, three authoring tools, and templates, as shown in Figure 4. When a BCI serious game solution is developed based on HNM, it should allow a diverse range of BCI serious games to be easily and quickly developed. In other words, the BCI serious game solution can be used repeatedly after it has been developed. Furthermore, as BCI serious game solutions include a variety of authoring tools, they reduce the overhead required for developing BCI serious games. The BCI serious game solution is built on BCI devices, so we consider these devices as the bottom layer of the solution. Next, there are a series of drivers for the BCI devices. During a game play, various BCI devices will be used, and these must be systematically managed and controlled. The solution manages and processes BCI devices using the BCI framework [2], which processes brainwaves using a unified interface, regardless of the BCI device type. Three authoring tools are developed based on this framework. Because authoring tools and BCI serious games work on the basis of the BCI framework, games can be designed and programmed independently of which types of BCI device are to be utilized.

In this implementation, we develop the BCI solution in C++ and the Win32 API in a Windows environment. This is because the moderate BCI devices such as Emotiv EPOC and Neurosky Mindset, BCI drivers, and BCI framework used in this implementation easily support the Windows environment.

### 4.2. BCI Framework

**Figure 5.**
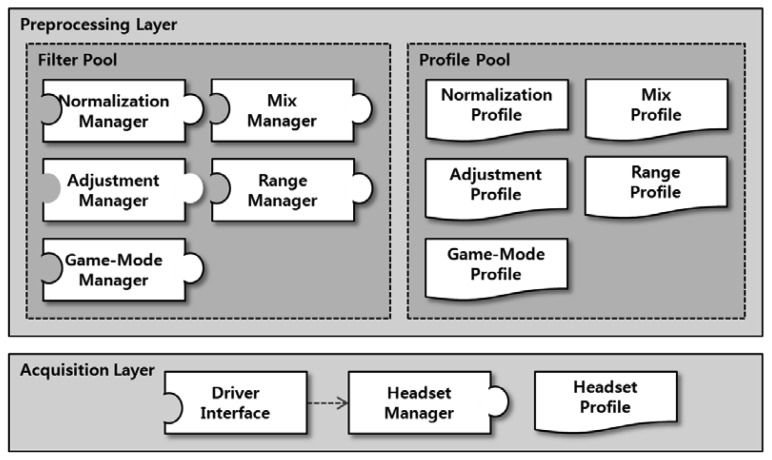
Structure of the BCI framework. acquisition layer unites BCI driver interfaces and preprocessing layer controls brainwaves.

The BCI framework specifies the interface for providing brainwaves, regardless of the BCI device type. Moreover, it also provides additional functions for simplifying the measurement of brainwaves. The framework is comprised of two layers, as shown in Figure 5.

The Acquisition Layer integrates BCI drivers from diverse third parties, and so provides the unified interface. The Preprocessing Layer converts the measured brainwaves so that they can be easily applied in games or applications. For example, the measured brainwaves are fed into the Normalization Manager, which outputs a distribution suitable for game control signals. The Mix Manager generates new wave patterns by combining more than one brainwave, and the Adjustment and Range Managers adjust and process the input brainwave patterns. Finally, the Game-Mode Manager increases or decreases the intensity of brainwaves depending on the difficulty level of the game. Through the functions described above, the Preprocessing Layer can reduce the cost and time required for BCI game development. Each profile specifies the option for the corresponding Manager. For example, the Adjustment Profile contains information on how much the brainwave has increased or decreased.

### 4.3. Authoring Tools

**Figure 6.**
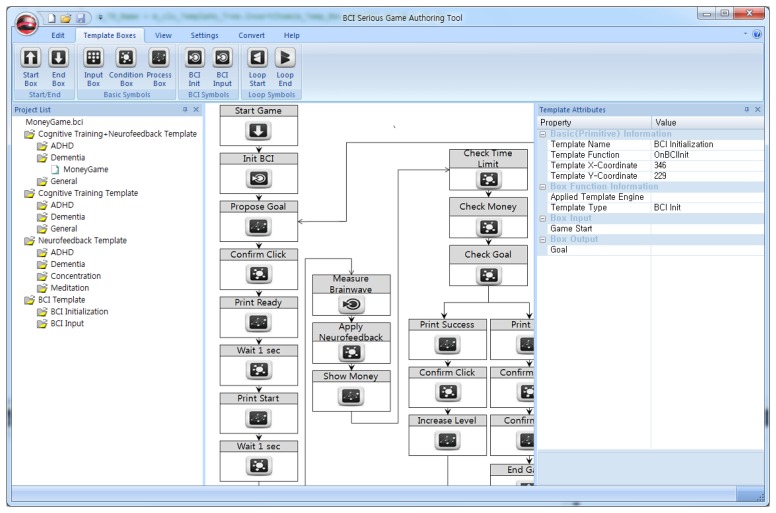
Authoring tool for game designers. game designer authoring tool provides the interface for drawing the flowchart of the BCI serious game.

Figure 6 shows the authoring tool used by experts, game designers, and game programmers. It provides a tree view to select and use templates, which are listed by category. The categories are BCI templates, neurofeedback templates, cognitive training templates, and cognitive neurofeedback templates. The template tree is only used by experts and game designers. The authoring tool also includes an interface for drawing flow charts, and a list of the properties denoted by symbols in the charts. In each template, the flow chart is described using the functions provided by authoring tools. In addition, only the expert authoring tool has the authority to create new templates. The game programmers authoring tool is only utilized for generating game codes.

### 4.4. Programming Process

After BCI codes and modules are generated in the list of functions corresponding to symbols prepared by experts and game designers, a set of Callback functions of the symbols in a flow chart are defined by game programmers. Then, the game programmers develop BCI serious games by integrating game engines from third parties on the basis of the BCI codes and modules.

**Figure 7.**
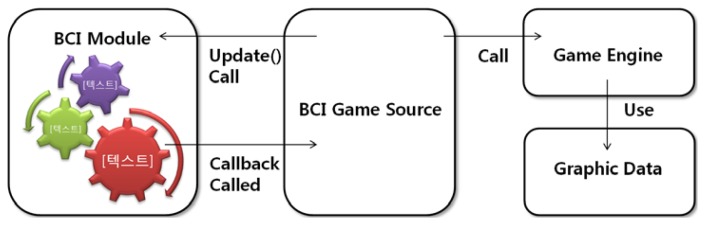
Program source diagram. The BCI module controls events via the update function.

Figure 7 shows the process for calling a Callback function in detail. First, when the developed BCI serious games are executed, the callback functions are registered by calling the functions of BCI modules, which define them for the purpose of connecting functions defined by programmers. Second, at regular intervals, the Update function of the BCI modules is called by the game programmers' code. Third, callback functions are automatically called by BCI modules in accordance with the flow chart. Some callback functions are called with brainwave values as parameters. Thus, game programmers can develop BCI serious games without technical knowledge of handling brainwaves, because the game is developed using transferred values without consideration of which brainwaves or features to use.

## 5. BCI Serious Game Development

This section describes the BCI game development process for patients with mild cognitive impairment using the BCI solution. First, the experts design and verify their templates for counting numbers. Next, we consider the approach taken by game designers to define the game design template based on the expert templates. Finally, we describe the BCI codes and modules generated by the game programmers.

### 5.1. Expert Template for a BCI Serious Game

**Figure 8.**
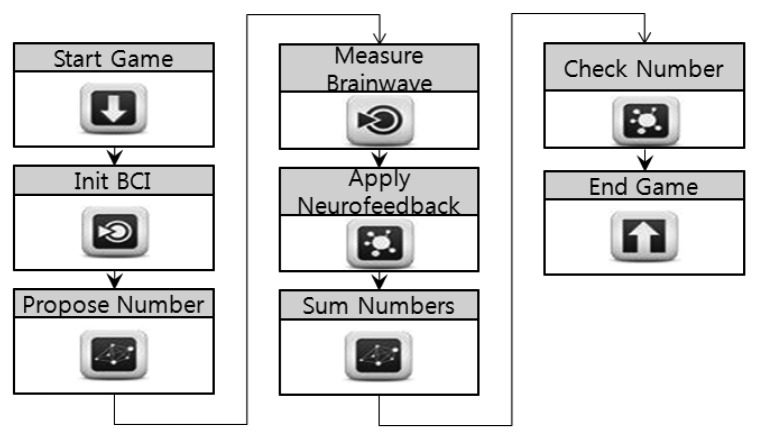
Expert Template for the Number Game. The flowchart contains the information of handling brainwaves and basic game processes.

The experts design a number counting game for the patients. A Sensory Motor Rhythm (SMR) wave is applied, which is converted into values from 0 to 100 by the BCI framework. This setting is described at the “Init BCI” symbol by experts. The goal of the game is to make a number by adding or subtracting values. Users must increase their brainwaves to 90 during the “Apply Neurofeedback” step as shown in Figure 8. When the converted value reaches 90, the patient can start to calculate numbers. The calculated numbers are checked and then the game ends. These rules are described in each symbol's property. By playing this game repeatedly, mathematical ability can be improved through summing numbers, and brain functions can also be improved through neurofeedback.

### 5.2. Design Template for a BCI Serious Game

We consider an experimental money game based around calculating the sum of numbers in the expert template. This game presents a target amount at the start of each stage. Users then collect different denominations of money that fall from the top of the screen in order to match the target amount. As the degree of difficulty increases, the target amount requires a more diverse set of denominations to be collected. When the amount collected exceeds the target amount, the user must return the sum to zero by catching a “bankruptcy” box. In addition, a “×2” box doubles the amount collected.

**Figure 9.**
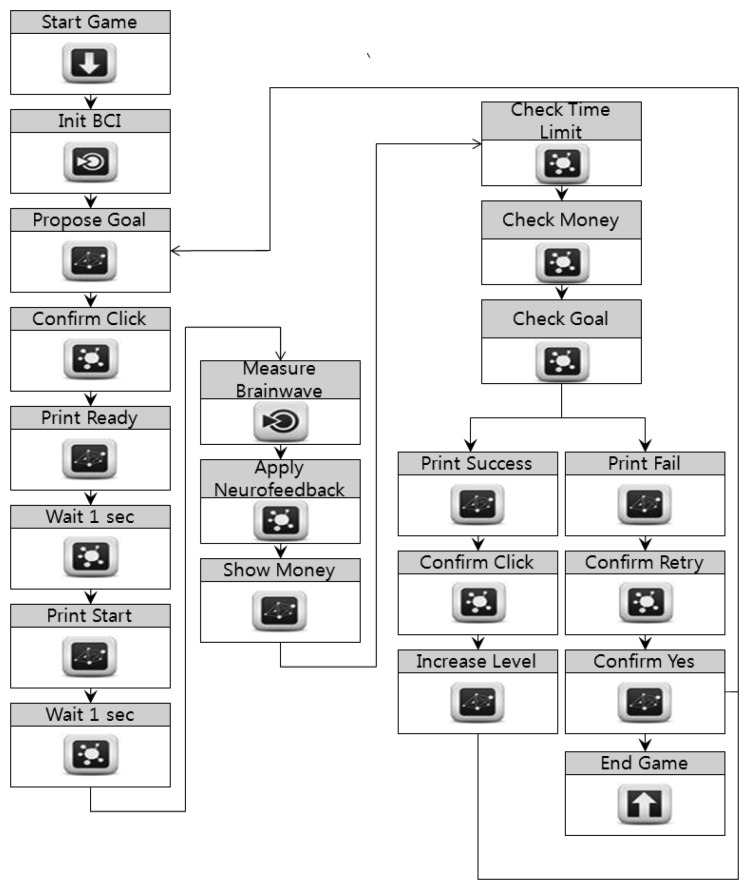
Game Design Template for the Money Game. BCI devices are initialized and a goal is proposed before the game starts.

The game is configured using the game designer authoring tool. Figure 9 shows the three-part flow chart of the game design template. First, the BCI devices are initialized and the goal of the game is proposed. “Start Game” shows the start window, and “Init BCI” initializes the BCI devices. The target amount depends on the user's level. After confirming the target amount, the user waits for the game to start, and then for the money to appear on the screen.

When the game starts, “Measure BCI” measures the user's brainwaves and applies neurofeedback. If the SMR wave does not maintain a value of over 90, the game is discontinued. In “Show Money,” the money begins to fall and the user collects various amounts by moving a basket with fingers on touchscreen or mouse or keyboard, to receive the money. Finally, the game state is evaluated and controlled. The target amount is checked after certain time intervals, as well as when a stage is successfully completed. When a stage is completed, a message of congratulations is displayed and the user acknowledges this success. The game level then goes up and a target amount is again presented. In case of failure, the game asks if the user wishes to play the game again. The user then confirms the failure and selects whether to continue the game. If the user stops the game, the application is closed. If the user continues the game, the level is not changed and a different target amount is again presented.

### 5.3. Generation of Game Codes and Modules for a BCI Serious Game

After the experts and game designers have completed their templates, the game programmer authoring tool converts them into BCI codes and modules. Table 6 shows the functions generated by the symbols in the flow chart in Figure 9. For example, the “Start Game” symbol generates the “OnStartGame” function in the BCI code. This function calls up OnStartGameCallback, the callback function registered in advance. “Init BCI” initializes the BCI devices, and does not require callback functions in the modules or the program source code. The symbol “Apply Neurofeedback” is the same. Unlike other functions, the symbol “Measure Brainwave” generates a function that receives an integer value. This is the value of a brainwave, as defined by an expert, among those measured by the BCI devices. The programmer uses this value to control the game.

### 5.4. Integration for a BCI Serious Game

**Figure 10.**
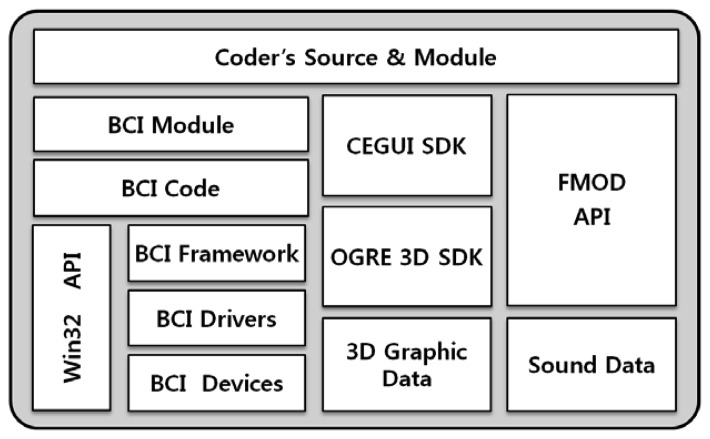
Relationship between BCI Code and Modules. BCI serious games are developed using BCI code, a BCI module, and diverse engines.

The generated BCI codes and modules are integrated with the programmer's source code and a number of engines, as shown in Figure 10. The BCI codes and modules are automatically generated by the Win32 API and the Open Game Rendering Engine (OGRE), which is introduced for 3D graphics processing. Next, the CEGUI software development kit is applied in OGRE for interface processing, and finally the FMOD API is applied for sound processing.

BCI serious games work on the basis of BCI devices, drivers, and frameworks, and also on the basis of the expert and game design templates that generate the BCI code and modules. Game programmers create program source code to complete a game by integrating third-party engines, BCI code, BCI modules, and data from graphical designers.

**Figure 11.**
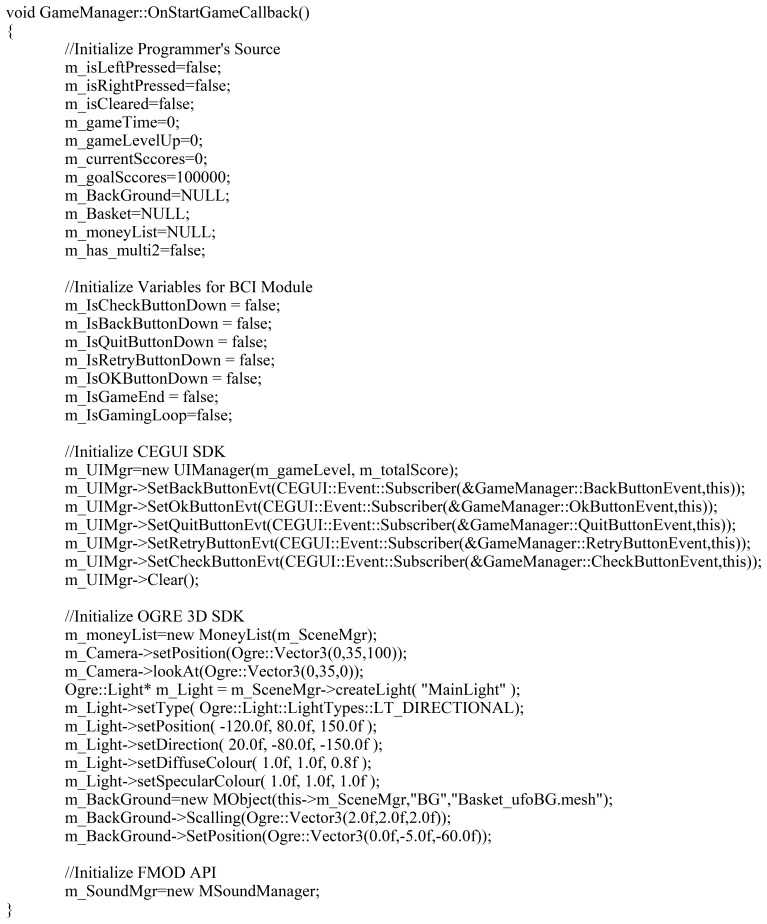
Implementation of Function OnStartGameCallback. This function initializes the money game.

**Figure 12.**
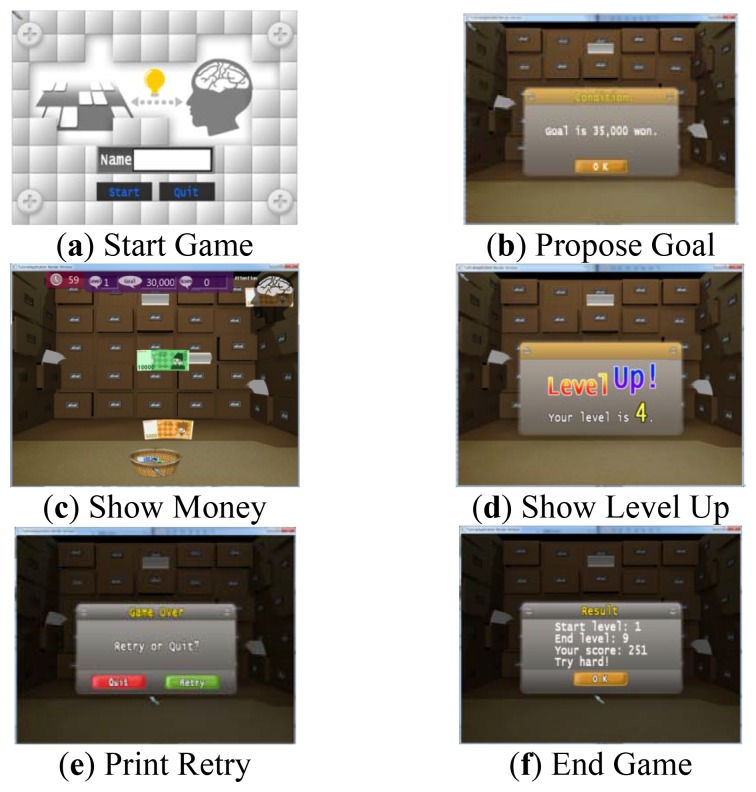
Money game play screen. Game is played according to the flowchart defined by experts and game designers.

The game is completed by implementing the Callback functions defined in Table 6. For example, OnStartGameCallback is implemented as shown in Figure 11. The game is also built using C++, and the function OnStartStartCallback is defined as a member function of the class GameManager. The function OnInitBCI is called, and the game engine is initialized. As described above, the programmer only implements Callback functions that will automatically be called from BCI modules. Figure 12 shows the BCI serious game developed by our approach.

## 6. Conclusions

This paper proposed a methodology for BCI serious game development. We defined the roles of experts, developers, and third parties in the development process. The key technology in BCI serious game development is the definition of expert templates, which require the most resources. We reduced the development cost of multiple BCI serious games by defining and reusing experts' knowledge in a series of templates. Moreover, by introducing authoring tools, experts on brainwaves, cognition, and brain functions could describe their technical knowledge by means of flow charts, which are easily understood by game designers and game programmers. Furthermore, the design and development of games is simplified by these expert templates. The development period was reduced because programmers could generate the BCI codes and modules required for development using authoring tools.

Future research will focus on a methodology that enables experts to describe their technical knowledge more easily, and the generation of templates based on that methodology to describe knowledge without requiring experts on brainwaves or cognition.

## Figures and Tables

**Table 1.** Output from experts.

**Workers**	**Work**	**Output**
Doctor, Brainwave Expert, & Cognitive Expert	Prepare the game development guide of BCI serious games, which can improve brain function.	Expert Template

**Table 2.** Output from third parties.

**Workers**	**Work**	**Output**
BCI Device Developer	Develop BCI Device and driver	BCI Device BCI Driver
Framework Developer	Develop BCI frameworksupporting a variety of BCI devices handling measured brainwaves	BCI Library BCI Engine BCI Framework
Game Engine Developer	Develop and supply 3D engines or sound engines required for BCI serious game development	3D Engine Sound Engine Network Engine

**Table 3.** Output from game developers.

**Workers**	**Work**	**Output**
Game Designer	Design the games based on the guide defined by all experts	Game Design Template
Graphic Designer	Design the graphic data required for a BCI serious game	Graphic Data
Game Programmer	Develop the game using BCI Framework and Graphic Data based on designed game	BCI serious game

**Table 4.** BCI symbols.

**Function**	**Description**
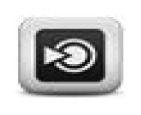 Initialize BCI	Initialize BCI device, then set parameters of brainwaves, which will be utilized to BCI serious game
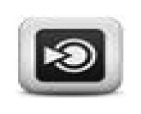 Measure brainwaves	Measure brainwaves from initialized BCI device

**Table 5.** Flow symbols.

**Function**	**Description**
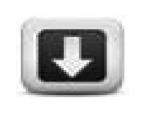 Start Game	Denote the position of game starting
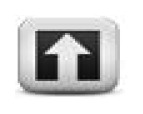 End Game	Denote the position of game ending
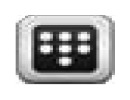 Input	Receive the input of a keyboard or a mouse
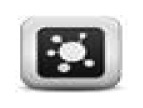 Check Condition	Denote the branch of a game
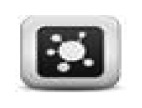 Process Functions	Denote the process position
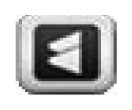 Start Loop	Denote the starting position of the repeatedly processed range
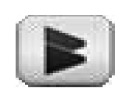 End loop	Denote the ending position of the repeatedly processed range

**Table 6.** Function Relationship.

**Symbol drawn by authoring tool in template**	**Function generated by authoring tool in BCI code & BCI module**	**Callback Function coded by programmer in programmer's program source**
Start Game	void OnStartGame()	void OnStartGameCallbackCallback()
Init BCI	void OnInitBCI()	-
Propose Goal	void OnProposeGoal()	void OnProposeGoalCallback()
Confirm Click	void OnConfirmClick()	void OnConfirmClickCallback()
Print Ready	void OnPrintReady()	void OnPrintReadyCallback()
Wait 1Sec	void OnWait1SecA()	void OnWait1SecACallback()
Print Start	void OnPrintStart()	void OnPrintStartCallback()
Wait 1Sec	void OnWait1SecB()	void OnWait1SecBCallback()
Measure Brainwave	OnMeasureBrainwave(int nBrainwave)	void OnMeasureBrainwaveCallback(int nBrainwave)
Apply Neurofeedback	void OnApplyNeurofeedback()	void OnApplyNeurofeedbackCallback()
Show Money	void OnShowMoney()	void OnShowMoneyCallback()
Check Time Limit	void OnCheckTimeLimit()	void OnCheckTimeLimitCallback()
Check Money	void OnCheckMoney()	void OnCheckMoneyCallback()
Check Goal	void OnCheckGoal()	void OnCheckGoalCallback()
Print Success	void OnPrintSuccess()	void OnPrintSuccessCallback()
Confirm Click	void OnConfirmClick()	void OnConfirmClickCallback()
Increase Level	void OnIncreaseLevel()	void OnIncreaseLevelCallback()
Print Fail	void OnPrintFail()	void OnPrintFailCallback()
Confirm Retry	void OnConfirm Retry()	void OnConfirm RetryCallback()
Confirm Yes	void OnConfirmYes()	void OnConfirmYesCallback()
End Game	void OnEndGame()	void OnEndGameCallback()
